# Alike but different: the evolution of the *Tubifex tubifex* species complex (Annelida, Clitellata) through polyploidization

**DOI:** 10.1186/1471-2148-14-73

**Published:** 2014-04-02

**Authors:** Roberto Marotta, Angelica Crottini, Elena Raimondi, Cristina Fondello, Marco Ferraguti

**Affiliations:** 1 Istituto Italiano di Tecnologia (IIT), via Morego 30, Genova 16163, Italy; 2 CIBIO, Centro de Investigação em Biodiversidade e Recursos Genéticos, Campus Agrário de Vairão, R. Padre Armando Quintas, Vairão 4485-661, Portugal; 3 Università degli Studi di Pavia, Dipartimento di Genetica e Microbiologia "A. Buzzati Traverso", Via Ferrata 1, 27100 Pavia, Italy; 4 Universitá degli Studi di Milano, Dipartimento di Bioscienze, Via Celoria 26, 20133 Milano, Italy

**Keywords:** *Tubifex*, Polyploidy, Speciation, Cryptic species, Reproduction, Cytogenetics, Molecular phylogenetics

## Abstract

**Background:**

*Tubifex tubifex* is a widespread annelid characterized by considerable variability in its taxonomic characteristics and by a mixed reproductive strategy, with both parthenogenesis and biparental reproduction. In a molecular phylogenetic analysis, we detected substantial genetic variability among sympatric *Tubifex* spp. from the Lambro River (Milano, Italy), which we suggested comprise several cryptic species. To gain insights into the evolutionary events that generated this differentiation, we performed a cytogenetic analysis in parallel with a molecular assay. Approximately 80 cocoons of *T. tubifex* and *T. blanchardi* were collected and dissected. For each cocoon, we sequenced a fragment of the 16S rRNA from half of the sibling embryos and karyotyped the other half. To generate a robust phylogeny enabling the reconstruction of the evolutionary processes shaping the diversity of these sympatric lineages, we complemented our original 16S rRNA gene sequences with additional COI sequences.

**Results:**

The chromosome number distribution was consistent with the presence of at least six sympatric euploid chromosome complements (one diploid, one triploid, three tetraploids and one hexaploid), as confirmed by a FISH assay performed with an homologous 18S rDNA probe. All the worms with 2n = 50 chromosomes belonged to an already identified sibling species of *T. tubifex*, *T. blanchardi*. The six euploid sets were coherently arranged in the phylogeny, with each lineage grouping specimens with the same chromosome complement.

**Conclusions:**

These results are compatible with the hypothesis that multiple polyploidization events, possibly enhanced by parthenogenesis, may have driven the evolution of the *T. tubifex* species complex.

## Background

Polyploidy is an important evolutionary mechanism in several taxa, both as a remote mechanism involving ancient genome duplications followed by extensive genetic reshufflings [1] and as a proximal cause of evolution (speciation by polyploidization) as, for example, in flowering plants [2,3] and, to a lesser extent, in animals [4]. Polyploids are often reproductively isolated from their diploid ancestors, as hybrids are anortoploid, producing unbalanced gametes at meiosis [4,5]. Various polyploidization events occurred in the remote past in vertebrates [1], and polyploidy has also been demonstrated among invertebrates, often in combination with parthenogenesis or other forms of asexual reproduction [4], thus promoting speciation. Among annelids, polyploidy is widely present in oligochaetous clitellates, with substantial differences within families [6,7].

*Tubifex tubifex* (Müller, 1774) is a cosmopolitan naidid annelid *sensu*[8] representing one of the major components of the benthic fauna in freshwater communities [9]. Also present in polluted waters, *T. tubifex* is widely used in laboratories for ecotoxicology research [10] and as a model organism for the study of annelid development [11]. *Tubifex tubifex* is characterized by considerable variability in its morphological features [12] and by a mixed reproductive strategy, with parthenogenesis [13], self-fertilization [14], and biparental reproduction through cross-mating [15]. In a molecular phylogenetic analysis of several *T. tubifex* and *T. blanchardi* specimens from the Lambro River (an intensively studied site in Northern Italy) based on a fragment of the 16S rRNA mitochondrial gene, we characterized *T. blanchardi*, traditionally considered a *T. tubifex "*morphotype" [9], and confirmed its status as a distinct species by an in-depth molecular and morphological analysis [16,17]. Moreover we detected considerable genetic variability among the "*T. tubifex"* specimens analyzed, and we suggest that these specimens may represent at least two cryptic species [16]. This study is in continuity with our previous work; thus, here, we use the same clade and lineage attributions.

 There are no cytogenetic data for *T. blanchardi* and few scattered records of *T. tubifex* due to the small size and large number of their chromosomes. The available cytogenetic data suggest that the observed morphological and molecular heterogeneity of *T. tubifex* is mirrored by numerous karyotypic differences [6]. Indeed, the different chromosome numbers proposed for *T. tubifex* (2n = 48, 2n = 75, 2n = 100, 2n = 110; 2n = 125; 2n = 150) might reflect different levels of ploidy within populations [6]. In agreement with these data, the presence of tetra- and hexaploid individuals among the *T. tubifex* inhabiting the Lambro River has been postulated [18] based on an analysis of the allozymes phosphoglucose isomerase (PGI) and phosphoglucose mutase (PGM). 

The aim of this study was to investigate polyploidy in sympatric *Tubifex* lineages coupling cytogenetics with molecular phylogenetics, and discuss how multiple polyploidyzation events, possibly enhanced by parthenogenesis, might have shaped the evolution of this species complex.

## Results

### Molecular identification and phylogenetic analysis

Seven indels, 122 variable sites (26.99%), and the empirical base frequencies πA = 0.319, πC = 0.214, πG = 0.207, and πT = 0.258 were identified in the mitochondrial 16S-rRNA gene alignment of the screened *T. tubifex* and *T. blanchardi* embryos, among which 16 haplotypes were identified.

Sequences were compared to the known *T. tubifex* and *T. blanchardi* haplotypes from the Lambro River (see Table 1). Building on a previously published analysis [16], seven new haplotypes were identified, but none corresponded to a potentially new genetic lineage and/or clade. Some of the material used by [16] has been recently reanalyzed (both molecularly and morphologically), thus allowing for the attribution of two of the haplotypes formerly assigned to the *T. tubifex* species complex (haplotype A11 and A12 in [16]) to another oligochaetous clitellate species, *Potamotrix bavaricus* ( [19]; personal observations).

**Table 1:** Samples analyzed

***Sample***	***Taxon***	***N° embryos for sequencing***	***N° embryos for cytogenetics***	***Haplotype***	***Clade***	***Lineage***	***Chromosome number***	***GenBank***	***Locality***
TAC138	*Tubifex blanchardi*	1	1	**-**	**-**	**-**	50	**-**	Lambro River, Italy
TAC139	*Tubifex blanchardi*	2	1	**-**	**-**	**-**	50	**-**	Lambro River, Italy
TAC140	*Tubifex blanchardi*	1	2	I	1	1	50	JQ247438	Lambro River, Italy
TAC141	*Tubifex tubifex*	3	2	VI	2	2a	75	JQ247439	Lambro River, Italy
TAC142	*Tubifex tubifex*	3	2	VI	2	2a	75	JQ247440	Lambro River, Italy
TAC143	*Tubifex tubifex*	2	1	VII	2	2b	100	JQ247441	Lambro River, Italy
TAC144	*Tubifex tubifex*	1	2	**-**	**-**	**-**	100	**-**	Lambro River, Italy
TAC145	*Tubifex tubifex*	2	1	XVI	2	2b	100	JQ247442	Lambro River, Italy
TAC146	*Tubifex tubifex*	2	1	VII	2	2b	100	JQ247443	Lambro River, Italy
TAC147	*Tubifex tubifex*	1	2	XIX	3	3a	100	JQ247444	Lambro River, Italy
TAC148	*Tubifex tubifex*	1	1	XI	3	3a	100	JQ247445	Lambro River, Italy
TAC149	*Tubifex tubifex*	2	2	VI	2	2a	75	JQ247446	Lambro River, Italy
TAC150	*Tubifex tubifex*	2	2	VII	2	2b	**-**	JQ247447	Lambro River, Italy
TAC151	*Tubifex tubifex*	3	2	XIX	3	3a	100	JQ247448	Lambro River, Italy
TAC152	*Tubifex tubifex*	1	2	XVIII	3	3b	100	JQ247449	Lambro River, Italy
TAC153	*Tubifex tubifex*	2	1	XVIII	3	3b	100	JQ247450	Lambro River, Italy
TAC154	*Tubifex tubifex*	2	2	**-**	**-**	**-**	100	**-**	Lambro River, Italy
TAC155	*Tubifex tubifex*	2	1	VII	2	2b	100	JQ247451	Lambro River, Italy
TAC156	*Tubifex tubifex*	1	1	XVIII	3	3b	100	JQ247452	Lambro River, Italy
TAC157	*Tubifex tubifex*	2	1	VII	2	2b	100	JQ247453	Lambro River, Italy
TAC158	*Tubifex tubifex*	2	1	XVIII	3	3b	100	JQ247454	Lambro River, Italy
TAC159	*Tubifex tubifex*	2	2	VII	2	2b	100	JQ247455	Lambro River, Italy
TAC160	*Tubifex tubifex*	2	1	XVIII	3	3b	100	JQ247456	Lambro River, Italy
TAC161	*Tubifex tubifex*	5	2	XIV	2	2c	150	JQ247457	Lambro River, Italy
TAC162	*Tubifex tubifex*	4	3	XIV	2	2c	150	JQ247458	Lambro River, Italy
TAC163	*Tubifex tubifex*	1	1	XIX	3	3a	100	JQ247459	Lambro River, Italy
TAC164	*Tubifex tubifex*	3	1	VII	2	2b	100	JQ247460	Lambro River, Italy
TAC165	*Tubifex tubifex*	3	1	XIV	2	2c	150	JQ247461	Lambro River, Italy
TAC166	*Tubifex tubifex*	2	2	XIV	2	2c	**-**	JQ247462	Lambro River, Italy
TAC168	*Tubifex tubifex*	1	1	XVI	2	2b	100	JQ247463	Lambro River, Italy
TAC169	*Tubifex tubifex*	5	2	XIV	2	2c	150	JQ247464	Lambro River, Italy
TAC170	*Tubifex tubifex*	2	1	VII	2	2b	100	JQ247465	Lambro River, Italy
TAC171	*Tubifex tubifex*	2	2	XVII	3	3b	100	JQ247466	Lambro River, Italy
TAC172	*Tubifex tubifex*	3	2	XIV	2	2c	150	JQ247467	Lambro River, Italy
TAC173	*Tubifex tubifex*	2	1	XII	3	3a	100	JQ247468	Lambro River, Italy
TAC174	*Tubifex tubifex*	2	2	**-**	**-**	**-**	75	**-**	Lambro River, Italy
TAC175	*Tubifex tubifex*	2	1	VII	2	2b	100	JQ247469	Lambro River, Italy
TAC176	*Tubifex tubifex*	2	1	XIV	2	2c	150	JQ247470	Lambro River, Italy
TAC177	*Tubifex tubifex*	1	1	VII	2	2b	100	JQ247471	Lambro River, Italy
TAC178	*Tubifex tubifex*	2	2	X	3	3a	100	JQ247472	Lambro River, Italy
TAC179	*Tubifex tubifex*	7	3	XIV	2	2c	150	JQ247473	Lambro River, Italy
TAC180	*Tubifex blanchardi*	2	2	I	1	1	**-**	JQ247474	Lambro River, Italy
TAC181	*Tubifex blanchardi*	1	1	I	1	1	50	JQ247475	Lambro River, Italy
TAC182	*Tubifex tubifex*	1	1	VI	2	2a	75	JQ247476	Lambro River, Italy
TAC183	*Tubifex tubifex*	1	1	VII	2	2b	100	JQ247477	Lambro River, Italy
TAC184	*Tubifex tubifex*	2	2	VII	2	2b	100	JQ247478	Lambro River, Italy
TAC185	*Tubifex tubifex*	2	1	VII	2	2b	100	JQ247479	Lambro River, Italy
TAC186	*Tubifex tubifex*	1	2	VI	2	2a	75	JQ247480	Lambro River, Italy
TAC187	*Tubifex tubifex*	2	1	XVIII	3	3b	100	JQ247481	Lambro River, Italy
TAC188	*Tubifex tubifex*	2	1	XIX	3	3a	100	JQ247482	Lambro River, Italy
TAC189	*Tubifex tubifex*	2	2	XII	3	3a	100	JQ247483	Lambro River, Italy
TAC190	*Tubifex tubifex*	2	2	VI	2	2a	75	JQ247484	Lambro River, Italy
TAC191	*Tubifex tubifex*	2	1	VII	2	2b	100	JQ247485	Lambro River, Italy
TAC192	*Tubifex tubifex*	2	1	VII	2	2b	100	JQ247486	Lambro River, Italy
TAC193	*Tubifex tubifex*	4	2	XIV	2	2c	150	JQ247487	Estonia
TAC194	*Tubifex tubifex*	5	2	XIV	2	2c	150	JQ247488	Estonia
TAC195	*Tubifex blanchardi*	2	1	I	1	1	50	JQ247489	Lambro River, Italy
TAC196	*Tubifex blanchardi*	2	1	I	1	1	**-**	JQ247490	Lambro River, Italy
TAC197	*Tubifex tubifex*	4	4	VI	2	2a	75	JQ247491	Lambro River, Italy
TAC198	*Tubifex tubifex*	2	2	VI	2	2a	75	JQ247492	Lambro River, Italy
TAC199	*Tubifex tubifex*	1	1	VII	2	2b	100	JQ247493	Lambro River, Italy
TAC200	*Tubifex tubifex*	1	1	XVIII	3	3b	100	JQ247494	Lambro River, Italy
TAC201	*Tubifex tubifex*	1	1	XVIII	3	3b	100	JQ247495	Lambro River, Italy
TAC202	*Tubifex blanchardi*	3	2	I	1	1	50	JQ247496	Lambro River, Italy
TAC203	*Tubifex blanchardi*	2	1	I	1	1	50	JQ247497	Lambro River, Italy
TAC204	*Tubifex blanchardi*	2	1	I	1	1	**-**	JQ247498	Lambro River, Italy
TAC205	*Tubifex tubifex*	1	1	XIX	3	3a	**-**	JQ247499	Lambro River, Italy
TAC206	*Tubifex tubifex*	2	2	VI	2	2a	**-**	JQ247500	Lambro River, Italy
TAC207	*Tubifex tubifex*	3	2	VI	2	2a	75	JQ247501	Lambro River, Italy
TAC208	*Tubifex tubifex*	3	2	VI	2	2a	75	JQ247502	Lambro River, Italy
TAC209	*Tubifex tubifex*	3	2	VI	2	2a	75	JQ247503	Lambro River, Italy
TAC210	*Tubifex tubifex*	1	2	VI	2	2a	**-**	JQ247504	Lambro River, Italy
TAC211	*Tubifex tubifex*	3	2	VI	2	2a	**-**	JQ247505	Lambro River, Italy
TAC212	*Tubifex tubifex*	4	4	VI	2	2a	75	JQ247506	Lambro River, Italy
TAC213	*Tubifex tubifex*	3	3	XIV	2	2c	150	JQ247507	Estonia
TAC214	*Tubifex tubifex*	5	4	XIV	2	2c	150	JQ247508	Estonia
TAC215	*Tubifex tubifex*	2	2	XIV	2	2c	150	JQ247509	Estonia
TAC216	*Tubifex tubifex*	4	2	XIV	2	2c	150	JQ247510	Estonia
TAC217	*Tubifex tubifex*	5	3	XIV	2	2c	**-**	JQ247511	Estonia
27**-**T	*Tubifex tubifex*	3	4	II	2	2d	100	JQ247437	Lambro River, Italy

Haplotypes, clades and lineage attributions were as in [16].

List of samples (31 embryos from 10 cocoons of T. blanchardi and 140 embryos from 70 cocoons of T. tubifex) used in parallel for cytogenetic and molecular analyses, with reference to the taxon and the number of embryos used for 16S rRNA gene sequencing and metaphase preparation, 16S rRNA gene haplotype, clade and lineage attribution and number of chromosomes. GenBank accession numbers and locality are also provided.

**Figure 1:**
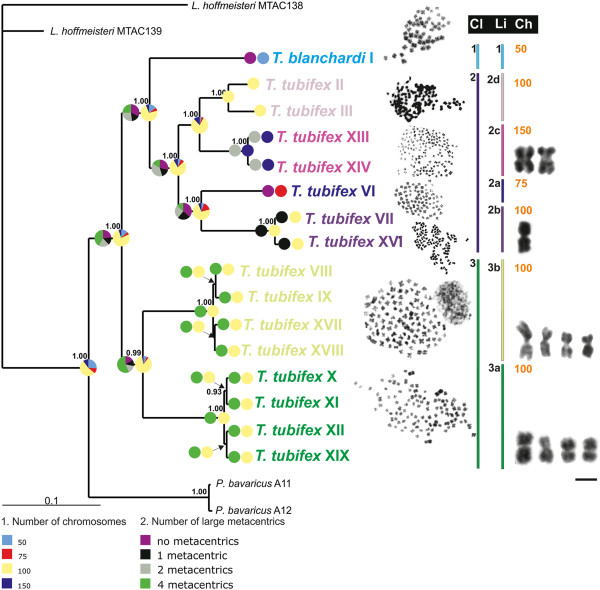
**Bayesian-inference phylogenetic tree of *T. tubifex *and *T. blanchardi *from the Lambro River, based on a combined 16S rRNA and COI genes data set, compared to representative metaphase plates of each lineage.** Representative images of the large metacentric-chromosome markers (four, two and one for, respectively, lineages 3a and 3b, lineage 2c and lineage 2b) are also shown. Bayesian posterior probabilities are indicated in front of the nodes. Only Bayesian posterior probabilities > 0.90 are shown. The ancestral state reconstruction from the Maximum-Likelihood Markov model (Mk1) describing the (1) number of chromosomes and (2) the number of large metacentric chromosomes is also reported on the topology. Pie diagrams at each node indicate the proportion of the Maximum Likelihood supporting alternative reconstructed states. Bars define clades and lineages. The roman numbers after the specie’s names identify the haplotypes as in Table 1. *Limnodirlus hoffmeisteri* MTAC138 and *L. hoffmeisteri* MTAC 139 were considered as outgroups. Ch, chromosome number; Cl, clade; Li, lineage. Scale bar is 2 μm.

The four partitioned Bayesian inference runs performed using both 16S rRNA and COI gene fragments (details below) identified 17 nodes, 13 of which were supported by posterior probabilities ≥ 0.99 in all runs (Figure 1). *Tubifex tubifex* and *T. blanchardi* haplotypes were organized in seven lineages. All *T. blanchardi* had an identical mitochondrial haplotype (Figure 1; Clade 1), while *T. tubifex* individuals comprised 15 different haplotypes, arranged in two well-supported major clades (Figure 1; Clades 2 and 3). Seven *T. tubifex* haplotypes (haplotypes: II, III, VI, VII, XIII, XIV, XVI) formed four well-supported lineages (see Figure 1; Lineages 2a-d), together being the sister group of the *T. blanchardi* clade (Figure 1; Clade 1 and Clade 2, 1.00 posterior probability values). Eight other haplotypes (haplotypes: VIII, IX, X, XI, XII, XVII, XVIII, XIX), assembled in two well supported lineages (see Figure 1; Lineages 3a-b), formed Clade 3 (0.99 posterior probability value), a sister group of the Clade1/Clade2 assemblage.

Within and between clades and lineages, the average uncorrected divergences at the 16S rRNA gene, summarized in Tables 2 and 3, were similar to those already published in [16]. Clades 2 and 3 showed a within-clade average uncorrected divergence (*p*-distance, transformed into percent using the complete delete option) of approximately 5% (see Table 2). The genetic distances between *T. blanchardi* and the other two clades were striking, ranging from 10.2% to 12.7%; by contrast, the genetic divergence between lineages of the same clade was low (see Table 3). A very low within-lineage genetic divergence (0.2% to 1.1%) characterized the *T. tubifex* lineages (see Table 2).

**Table 2:** Within clade (bold) and within lineage genetic divergence of the analysed 16S rRNA gene fragment

***T. blanchardi_1***	**n/c**
***T. tubifex_2***	**5.4%**
*T. tubifex_2a*	n/c
*T. tubifex_2b*	0.7%
*T. tubifex_2c*	0.2%
*T. tubifex_2d*	1.1%
***T. tubifex_3***	**5.0%**
*T. tubifex_3a*	0.6%
*T. tubifex_3b*	1.0%

The genetic divergence is based on the pairwise distance calculation (using the complete delete option) for the *T. blanchardi* and the different *T. tubifex* clades and lineages.

**Table 3:** Among clades (bold) and among lineages genetic divergence of the analysed 16S rRNA gene fragment

	***T. blanchardi_1***	***T. tubifex_2***	***T. tubifex_2a***	**T. tubifex_2b**	***T. tubifex_2c***	***T. tubifex_2d***	***T. tubifex_3***	***T. tubifex_3a***	***T. tubifex_3b***
*T. blanchardi_1*	**-**	**-**							
***T. tubifex_2***	**10.2%**	**-**							
*T. tubifex_2a*	10.1%	**-**	**-**						
*T. tubifex_2b*	9.8%	**-**	7.3%	**-**					
*T. tubifex_2c*	11.3%	**-**	6.2%	7.3%	**-**				
*T. tubifex_2d*	9.6%	**-**	6.7%	5.6%	5.0%	**-**			
***T. tubifex_3***	**12.7%**	**12.7%**	**-**	**-**	**-**	**-**	**-**		
*T. tubifex_3a*	12.6%	**-**	12.5%	12.5%	13.7%	13.0%	**-**	**-**	
*T. tubifex_3b*	12.7%	**-**	11.4%	12.8%	13.0%	12.1%	**-**	8.1%	**-**

The genetic divergence is based on the pairwise distance calculation (using the complete delete option) for the *T. blanchardi* and the different *T. tubifex* clades and lineages.

### Cytogenetic analysis

#### Chromosome-number distribution

**Figure 2:**
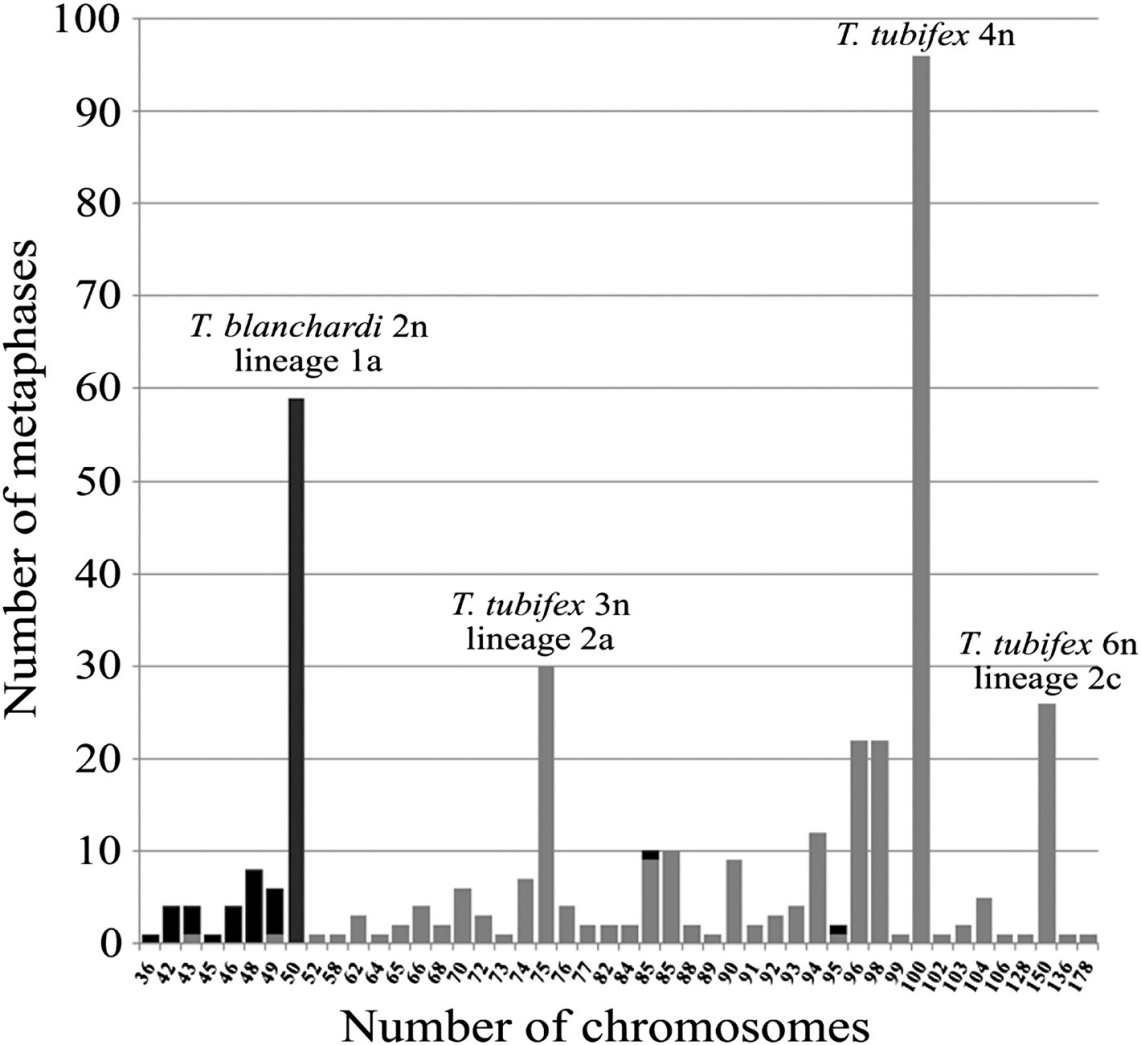
**Chromosome-number distribution.** Histogram showing the chromosome-number distribution in Tubifex blanchardi (black) and T. tubifex (gray).

Based on the analysis of more than 80 metaphase plates, *T. blanchardi* showed a unimodal distribution, with a modal chromosome number of 50. The chromosome-number distribution of the *T. tubifex*, based on the analysis of more than 290 metaphase plates*,* showed a trimodal pattern, with a primary mode of 100 chromosomes and secondary and tertiary modes of 75 and 150 chromosomes, respectively (Figure 2).

#### Karyotype reconstruction

**Figure 3:**
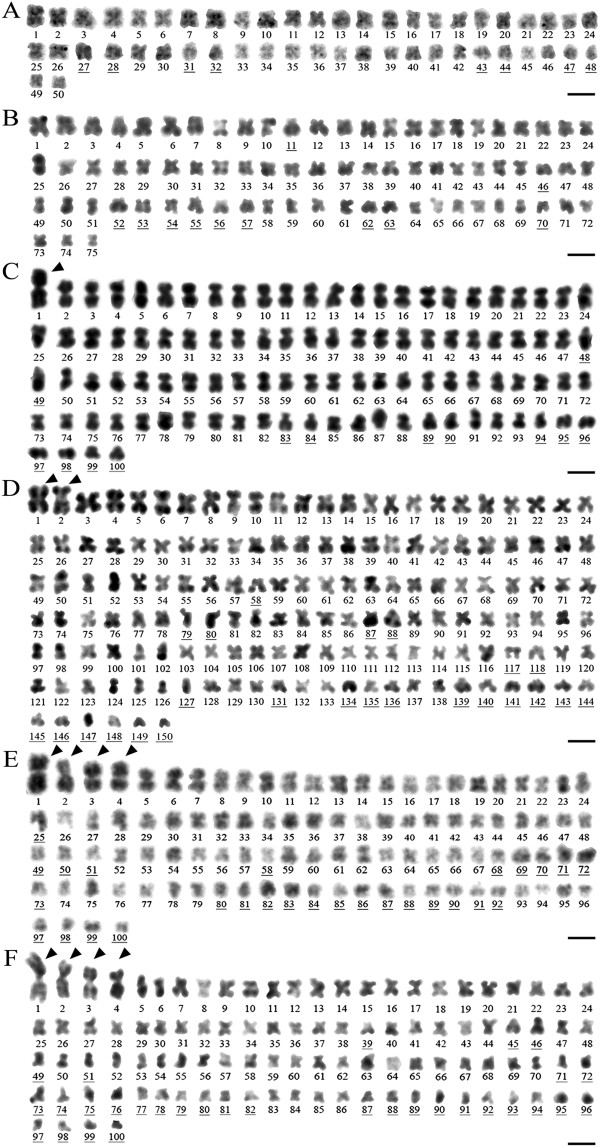
**Karyotype reconstruction on metaphase chromosomes of *T. blanchardi *and of the *T. tubifex *lineages of the Lambro River. A**. The diploid *T. blanchardi* (2n = 50), consisting of 42 meta- and submetacentric chromosomes and 8 acrocentric chromosomes; **B**. the triploid *Tubifex* (lineage 2a, 3n = 75) composed of 64 meta submetacentric chromosomes and 11 acrocentric chromosomes; **C**. the tetraploid *Tubifex* (lineage 2b, 4n = 100) composed of 87 meta-, submetacentric chromosomes and 13 acrocentric chromosomes, with one large metacentric chromosome marker (arrowhead); **D**. the hexaploid *Tubifex* (lineage 2c, 6n = 150), composed of 128 meta- submetacentric chromosomes and 22 acrocentric chromosomes, with two large metacentric chromosome markers (arrowheads); **E**. the tetraploid *Tubifex* (lineages 3a, 2n = 100) composed of 74 meta- or submetacentric and 26 acrocentric chromosomes, with four large metacentric chromosome markers (arrowheads); **F**. the tetraploid *Tubifex* (lineages 3b, 2n = 100) composed of 69 meta- or submetacentric chromosomes and 31 acrocentric chromosomes, with four large metacentric chromosome markers (arrowheads). Numbers indicate the meta- or submetacentric chromosomes; underlined numbers indicate acrocentric chromosomes. Scale bars are 4 μm in A, 3 μm in B, 2.5 μm in D and 2 μm in C, E, and F.

The GIEMSA-stained metaphase plates of *T. blanchardi* and *T. tubifex* were selected on the basis of spreading quality to minimize the number of artifacts due to loss or gain of single chromosomes. The chromosomes were aligned by decreasing size; on the basis of centromere position, they were classified as meta and submetacentric chromosomes or acrocentric chromosomes. It was not possible to reconstruct the karyotype of the rare *T. tubifex* lineage 2d due to the poor quality of the few metaphases observed. The size and relative number of meta- submeta- and acrocentric chromosomes for the representative karyotypes of the various lineages are summarized in Figure 3. One, two and four metacentric chromosomes significantly larger than the others were found in the metaphase plates of lineages 2b (one), 2c (two), 3a and 3b (four; Figures 1 and 3).

#### In-Situ Hybridization

**Figure 4:**
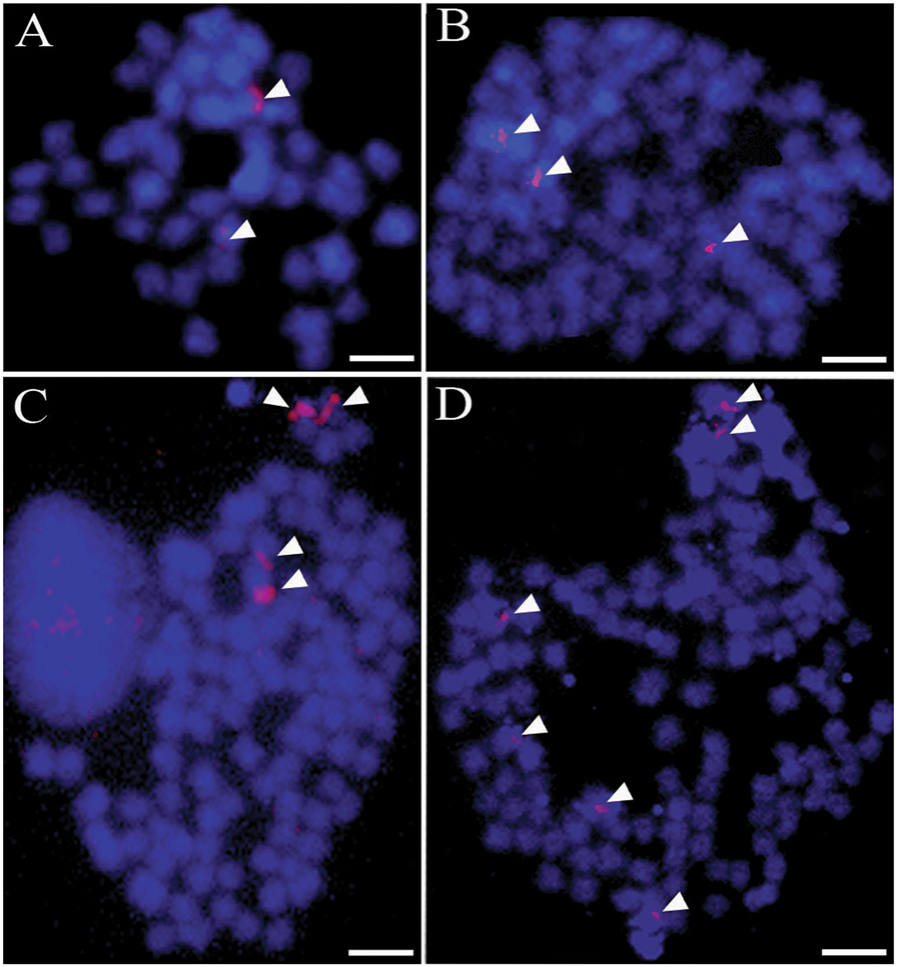
**FISH analysis of metaphase chromosomes of *T. blanchardi *(A) and the *Tubifex *lineages of the Lambro River with 75 (B), 100 (C) and 150 (D) chromosomes.** The chromosomes are pseudocolored in blue, and the 18S rRNA gene hybridization signals are pseudocolored in red (arrowheads). Scale bars are 4 μm.

Due to the large number and small size of the *T. tubifex* and *T. blanchardi* chromosomes, it was impossible to finely localize the 18S rDNA signals on specific chromosomes; we therefore simply counted the number of hybridization signals in each metaphase plate. All the analyzed metaphase plates from *T. blanchardi* showed two hybridization signals (Figure 4A), while *T. tubifex* lineage 2a (75 chromosomes) showed three hybridization signals (Figure 4B); lineages 2b, 2d, 3a and 3b (100 chromosomes), showed four hybridization signals (Figure 4C); and lineage 2c (150 chromosomes) showed six hybridization signals (Figure 4D).

## Discussion

The karyotype of *Tubifex blanchardi,* assessed here for the first time, differed from that of the other sympatric *T. tubifex* lineages, thus corroborating its status as a distinct species, as proposed by [16]. The observed chromosome numbers and size distribution were consistent with the presence of at least six sympatric euploid chromosome complements, corresponding to one diploid (n = 50, *T. blanchardi*), one triploid (n = 75), three tetraploids (n = 100) and one hexaploid (n = 150) *T. tubifex* populations. All 7 analyzed *T. tubifex* from Estonia were hexaploid (2n = 150) and grouped into lineage 2c, with other individuals from the Lambro River. The numbers of hybridization signals we observed in *T. blanchardi* and in the various *T. tubifex* lineages were consistent with the ploidy levels inferred from the analysis of the chromosome number distribution. The presence of large metacentric chromosomes proportionate in number to the ploidy level, as proposed by [6], has been verified only for the tetraploid *T. tubifex* belonging to Clade 3, which have four large metacentric chromosomes. Only one large metacentric chromosome was observed in the tetraploid lineage 2b, and two large metacentric chromosomes were observed in the hexaploid lineage 2c (see Figure 1). The large metacentric chromosomes might be derived from centric fusions of acrocentric chromosomes (Robertsonian translocation), as already reported in other families of oligochaetous clitellates (for example, among enchytraeids and lumbricids; [6]). By mapping the *T. tubifex* and *T. blanchardi* karyotypes on the phylogenetic tree, we identified at least two *T. tubifex* clades: the two tetraploid populations of Clade 3 and the tri-, tetra-, and hexaploid populations that form the well supported Clade 2. The *T. tubifex* of Clades 2 and 3 are characterized by a high interclade genetic divergence (more than 12%; see Table 3) suggesting the existence of a genetic barrier to gene flow [16], and should be considered distinct cryptic species. The different polyploid populations forming Clade 2 and Clade 3 may be considered polyploid forms of the same species (see, for example [20]) or distinct cryptic species. Intriguingly, the intraclade genetic divergence among 16S rRNA genes ranges from 5.0% to 8.1% (see Table 2), a value comparable to the genetic divergence observed between polyploid populations of plants recognized as true polyploid species [21]. Although a formal description of these lineages falls outside the aims of this study, we stress that it was not possible to identify any consistent morphological difference between Clades 2 and 3, or between the different identified lineages [16]. The morphological characteristics traditionally used for identification in this group allowed for the identification and characterization only of *T. blanchardi*[17]*;* however, following an integrative taxonomic approach [22], the genetic complements of the various lineages (this study), their independent evolutionary histories and, perhaps, their ecological features suggested by [23], might be considered good taxonomic markers to be used for a formal description of these independent lineages.

### *Evolution of the* Tubifex *species from the Lambro River*

The six euploid sets that we observed in the mixed Lambro community were coherently arranged in the mitochondrial phylogenetic tree, each lineage grouping specimens with the same chromosome complement (Figure 1). No lineages of mixed ploidy were observed, and each polyploid was represented by one or few mitochondrial haplotypes (see Table 1), as is common in parthenogenetic polyploid species [24]. Parthenogenesis may have played a key role in the evolution of polyploidy in *T. tubifex* and *T. blanchardi*, as in other clitellates [6,25] and in many animal groups [24,26]. This pattern, together with the pattern of hybridization signals, suggests that multiple polyploidyzation events (perhaps associated with parthenogenesis) may have played a driving role in maintaining independently evolving species and in shaping the evolution of the Lambro *T. tubifex* and *T. blanchardi* species.

Two main hypotheses concerning chromosomal evolution are compatible with our phylogenetic analysis. In the first scenario, a diploid (2n = 50) common ancestor may have given rise to clades 1 + 2 and 3 through two independent polyploidyzation events. Indeed, the tetraploid *T. tubifex* belonging to Clade 2 (lineages 2b and 2d) and Clade 3 differ in their karyotypes, respectively showing none, one and four large metacentric chromosomes and different proportions of meta-, submeta- and acrocentric chromosomes (Figures 1 and 3). Following this hypothesis, *T. blanchardi* retained the plesiomorphic diploid state of 50 chromosomes [6]. The alternative scenario, which is more likely according to our ancestral state reconstruction analysis, suggests that the ancestor of Clades 1 + 2 and 3 was instead tetraploid (Figure 1). Clade 3 may have arisen directly from this tetraploid ancestor, which probably had a similar karyotype, with four large metacentric chromosomes (Figure 1). By contrast, the diploid *T. blanchardi* (Clade 1) and the tetraploid *T. tubifex* (Clade 2; lineages 2b, 2d) evolved from a new tetraploid ancestor, characterized by a proportion of large metacentric chromosomes different from that seen in the ancestral population (Figures 1 and 3). Following this hypothesis, the diploid *T. blanchardi* (Clade 1) may have originated from a tetraploid secondarily, possibly by parthenogenetic development of its eggs (Figure 1). The triploid (Clade 2, lineage 2a; see Figure 1) and hexaploid *T. tubifex* lineages (Clade 2, lineage 2c; see Figure 1) might have originated from this tetraploid ancestor from the union of a haploid and an unreduced gamete or through hybridization among cryptic species.

Polyploid species generally have greater metabolic flexibility and are characterized by an ecological tolerance higher than that of their diploid ancestors ( [27]; also see [4,28] for a critical review). Although controversial, an example of environmental tolerance acquired via polyploidy concerns *Artemia parthenogenetica,* the polyploid populations of which are more likely to survive in polluted habitats, at suboptimal values of temperature and salinity or in the presence of elevated concentrations of cadmium, than their diploid ancestors [29]. Although several studies have demonstrated that different genetic lineages of *T. tubifex* have different habitat preferences and resistance to pollutants (see [23] and citations therein), no direct effect of ploidy on pollution resistance has been suggested. [23] showed that *T. tubifex* individuals belonging to different mitochondrial lineages varied consistently in cadmium resistance. Intriguingly, when the *T. tubifex* 16S rRNA gene sequences from [23] corresponding to the lineages with different levels of cadmium resistance were mapped against our phylogeny, they grouped on the phylogenetic tree in a predictable manner. Indeed, all the sequences belonging to lineages I, II, III, IV and V *sensu*[23] grouped with the triploid, tetraploid and hexaploid *T. tubifex* respectively belonging to lineages 3a, 2a, 2b, 2d and 2c of the Lambro River, excluding sequence D6-U9 (which officially belongs to lineage II; GenBank accession number AJ225905; Additional file 1: Figure S1). The cadmium-resistant lineages (I and III) corresponded to tetraploid lineages 3a and 2b, and the less cadmium-resistant of the two (lineage II) corresponded to the triploid *T. tubifex* lineage 2a (Additional file 1: Figure S1). The differences in ploidy in *T. tubifex* may thus help to explain the differences in ecological tolerance, although this analysis must be investigated further. Similarly, the strong connection between mitochondrial lineages and ploidy levels can be extended to *Tubifex* specimens from other regions worldwide.

## Conclusions

In this study, we propose alternative hypotheses for the evolution of the independent sympatric *T. tubifex* and *T. blanchardi* lineages, analyzing the chromosome-number distribution and karyotypes in relation to their phylogenetic relationships. Combining molecular phylogenetics and cytogenetics, we have found evidence for the existence of six different euploid chromosome sets coherently arranged in the *Tubifex* mitochondrial phylogeny, each lineage grouping specimens that share the same chromosome complement. Our results, including a FISH assay, suggest that our population comprise diploid, triploid, tetraploid and hexaploid sympatric individuals, and that the process of polyploidization, possibly enhanced by parthenogenesis, may have played an important role in both speciation and in the evolution of *T. tubifex* genetic lineages characterized by different resistance to pollutants. In the future, controlled breeding experiments should be performed with the different *T. tubifex* lineages to observe parthenogenesis and thus shed light on the reproductive strategies adopted by the different *Tubifex* lineages. Moreover, a phylogeny based on independent nuclear markers should allow us to understand if parthenogenesis and polyploidy are associated to interspecific hybridization.

## Methods

### Sampling and laboratory cultures

Specimens of *T. tubifex* and *T. blanchardi* were collected from a mixed naidid community in the Lambro River (Milano, Lombardy, Italy) between January 2006 and January 2009. Sexually mature individuals were identified under a light microscope according to the traditional taxonomic keys [30]. The worms were reared at low densities on a sterilized sand substrate covered with tap water, fed leaves of spinach, and kept at 20°C. Food and water were changed weekly. So that cytogenetic and molecular analyses could be performed in parallel, 70 cocoons of *T. tubifex* and 10 cocoons of *T. blanchardi* were collected from the reared worms and dissected. For each cocoon, about half of the embryos were used for molecular analyses and the remaining embryos were processed for cytogenetic analysis. All specimens came from the shallow lateral trickles of the Lambro River, with the exception of 7 individuals from Estonia (kindly provided by Tarmo Timm) that were used as a reference (see Table 1). The *T. tubifex* and *T. blanchardi* community in the Lambro River has been analyzed previously by [16] and populations of the lateral trickles at different areas and depths of the sampling sites where molecularly and morphologically uniform, also across seasons.

### Molecular analysis

#### DNA extraction and sequencing

DNA from one to three embryos for each cocoon was extracted, and the mitochondrial 16S rRNA gene was sequenced. Total genomic DNA was extracted from the tissue samples using proteinase-K digestion (10 mg/ml concentration), followed by a standard salt extraction. For primers and cycling protocols, see [16]. PCR products were loaded onto 1% agarose gels, stained with ethidium bromide, and visualized on a "Gel Doc" system (PeqLab). Bands of the correct size were purified using QIAquick spin columns (Qiagen) and sequenced on an automated sequencer, ABI 3130XL (Applied Biosystems, Perkin Elmer).

#### Sequence alignment and molecular identification

Sequences were confirmed by a BLAST search in GenBank, and chromatographs were checked by eye and edited, when necessary, using CodonCode (version 3.7.1; Codon Code Corporation). The alignment of all sequences required the inclusion of gaps to account for indels in some hypervariable regions. All newly determined sequences were submitted to GenBank (accession numbers: JQ247437-JQ247511; for details, see Table 1). All sequences obtained in this study were merged with all known haplotype sequences of *T. blanchardi* and *T. tubifex* from the Lambro River [16]. The number of base substitutions and the empirical frequencies of different nucleotides were determined using MEGA, version 5.05 [31]. Sequences were merged into haplotypes using the online application DNAcollapser v.1.0 ( http://www.birc.au.dk), resulting in 16 haplotype sequences.

#### Phylogenetic analyses

As phylogenetic analyses based on a single marker did not provide a robust phylogenetic hypothesis for the evolution of this species complex (see Figure 1 in [16]), and as no additional embryonic DNA was available, we molecularly characterized the mitochondrial 16S rRNA and COI gene fragments from additional 106 specimens of *T. tubifex* and *T. blanchardi*, 2 *Limnodrilus hoffmeisteri* and 2 *Potamotrix bavaricus* specimens [Genbank accession numbers EU117465-EU117509; EU117546-EU117547; EU311221-EU311273; EU311275-EU311285 (16S); EU311286- EU311382; EU311384-EU311398 (COI)], some of which were already available from [16]. The protocol described above was followed for DNA extraction and PCR amplification of the 16S rRNA gene fragment. We used the primers LCO1490 5’-GGT CAA CAA ATC ATA AAG ATA TTG G-3’ and HCO2198 5’-TAA ACT TCA GGG TGA CCA 9 AAA AA T CA-3’ [32] to amplify an approximately 650-bp fragment of the mitochondrial COI gene. PCRs were performed in 23 μl reactions using 1.5 μl of genomic DNA, 2 μl of each 10 pmol primer, 0.5 μl of total dNTPs (10 mmol in water; Promega), 0.1 μl of 5 U/μl GoTaq®, 5 μl 5× Green GoTaq® Reaction Buffer (Promega) and 11.9 μl of water. The PCR conditions were as follows: an initial denaturation step at 94°C for 90 seconds, 35 cycles of denaturation at 94°C for 30 seconds, annealing at 49°C for 45 seconds, and extension at 72°C for 90 seconds, with a final extension for 10 min at 72°C. PCR products were loaded onto 1% agarose gels, stained with ethidium bromide, and visualized on a "Gel Doc" system (PeqLab). Bands of the correct size were purified using QIAquick spin columns (Qiagen) and sequenced on an automated sequencer ABI 3130XL (Applied Biosystems, Perkin Elmer). Identity of the sequences was confirmed with a BLAST search in GenBank. Chromatographs were checked by eye and edited, when necessary, using CodonCode (version 3.7.1; Codon Code Corporation). The alignment of all sequences required the inclusion of gaps to account for indels in some hypervariable regions of the 16S rRNA gene fragment. The sequencing of these 110 samples enabled the identification of the same 16 *T. tubifex* and *T. blanchardi* haplotypes. These sequences, plus the four sequences of the outgroups *L. hoffmeisteri* and *P. bavaricus,* were combined with their corresponding COI sequences, and the resulting dataset was used to perform the phylogenetic analyses. The software Gblocks [33] was used to delete highly divergent regions that could not be unambiguously aligned or that were saturated by multiple substitutions. We surveyed a number of data-partitioning schemes using PartitionFinder [34]. Because the software required the user to pre-define partitions and specify them in the configuration file, we created an input configuration file that contained four partitions corresponding to individual codon positions in the COI gene fragment and the 16S rRNA gene fragment, which was the most finely partitioned scheme possible in our mitochondrial dataset. We used the "greedy" algorithm (heuristic search) with "unlinked" branch lengths in PartitionFinder to search for the best-fit and worst-fit Schemes. A total of seven *a priori* schemes with varying degrees of complexity were statistically compared in PartitionFinder. We used AIC, natively implemented in PartitionFinder, to compare partitioning schemes. The more complex partition strategy (four partitions) yielded the analysis with the lowest score and therefore was identified as the optimal partitioning scheme for our analyses. The SYM + G, F81, HKY + G and SYM + I + G models were identified in PartitionFinder as the best-fitting models of substitution for the 1-, 2- and 3-codon positions of the COI and the 16 s rRNA gene respectively.

Partition Bayesian analyses were performed using MrBayes 3.2.1 [35]. We performed four runs of 10 million generations (started on random trees) and four incrementally heated Markov chains (using default heating values), sampling the Markov chains at intervals of 1,000 generations. Convergence and mixing of chains in the Bayesian phylogenetic analyses were assessed by examining output files with the AWTY (Are We There Yet) graphical exploration software [36]. This tool confirmed that the split frequencies among runs were strongly correlated and that the topological differences between trees sampled by independent runs stabilized after ca. 1.5 million generations. Stabilization and convergence of likelihood values occurred after 2 million generations; therefore, the first 2 million generations were discarded as burn in, and eight million trees were retained and summed to generate the majority-rule consensus tree. Homologous sequences of *L. hoffmeisteri* and *P. bavaricus* were defined as outgroup.

#### Ancestral state reconstruction

Likelihood unequivocal reconstruction of traits, all treated as unordered was performed using the ancestral state module implemented in MESQUITE (version 2.75; [37]). Trait evolution and ancestral states were reconstructed by mapping each single trait on the rooted topology generated from the Bayesian analyses (Figure 1). The following morphological traits were considered in the reconstruction of evolutionary pathways: (1) number of chromosomes and (2) number of large metacentric chromosomes. As the chromosome number of *P. bavaricus* is unknown, we attributed to this species the chromosome complement (2n = 50) of *P. hammoniensis*[6]*.* For the maximum-likelihood analysis of chromosomal apomorphies and ancestral state reconstruction, we used the symmetrical Markov k-state one-parameter model (MK1). The likelihoods are reported as proportional likelihoods and are represented as pie charts in Figure 1.

### Cytogenetic analysis

Cocoons were collected 48 h after deposition and transferred to Petri dishes in CMF buffer [Calcium Magnesium Free solution], and the embryos were carefully dissected from their cocoons in a mitosis-rich developmental stage (192 to 288 h after fecundation). Embryos were processed for different assays as follows:

a) *Chromosome preparation*. More than 350 metaphase plates were prepared from 1 to 4 embryos of *T. blanchardi* and *T. tubifex* specimens, as described by [38]. The embryos were incubated in CMF buffer containing 0.5 mg/ml Colcemid (GIBCO) and, after a hypotonic treatment with a KCl 2 mM solution, were fixed with freshly prepared cold 3:1 methanol:acetic acid for 1 h. The embryos were placed on a microscope slide on a warm plate and covered with a drop of 60% acetic acid, and their cells were mechanically dispersed in the drop by tapping the tissue with the flat end of a scalpel. The preparations were stained with GIEMSA stain (10% GIEMSA Gurr’s R66) in Sörensen buffer, rinsed in distilled water, mounted with Sintex and observed under a Jenaval optical microscope.

b) *In-situ hybridization*. Fluorescence *in situ* hybridization (FISH) on metaphase chromosomes with rRNA probes has been carried out only in a few clitellate species to assess their ploidy level: the lumbricids *Eisenia foetida* and *Octodrilus complanatus*[39,40] and the hirudinean *Haemopis sanguisuga*[41]. Although those diploid clitellate species are not closely related, all showed FISH signals on a single chromosome pair for each metaphase plate, suggesting that the ribosomal genes are probably organized in a single multigenic cluster. More than 30 metaphase plates from *T. tubifex* and *T. blanchardi* embryos were prepared as described above and processed for FISH analysis. To prepare the homologous DNA probe for FISH experiments, total genomic DNA was isolated from approximately 100 *T. tubifex* and *T. blanchardi* specimens by standard phenol/chloroform extraction. The 18S rRNA gene fragments were amplified directly from genomic DNA by PCR using the primers TIMAF 5’-AMCTGGTTGATCCTGCCAG-3’ and TIMBR 5’- TGATCCATCTGCAGGTTCACCT-3’ [42]. PCR was performed with the following cycling protocol: 95°C for 5 seconds, followed by 20 cycles of 95°C for 30 seconds, 57°C for 30 seconds, and 72°C for 90 seconds, with a final elongation step at 72°C for 8 minutes. Approximately 100 ng of the 18S rRNA PCR product, purified using ammonium acetate and ethanol, was labeled with digoxigenin-11-dUTP by nick translation using the DIG-Nick translation kit (Roche). Each FISH experiment was performed in parallel on *T. tubifex*, *T. blanchardi* and human metaphase chromosome preparations (as a control for hybridization efficiency). After treatment with RNase A (Sigma-Aldrich), the slides were incubated for 30 seconds in 0.005% pepsin in 0.01 N HCl at room temperature, rinsed in PBS, denatured in 70% deionized formamide in 2XSSC for 2 minutes at 75°C, and then dehydrated in a 70%, 90% and 100% ethanol series for 2 minutes each. One hundred nanograms of labeled probe was denatured for 8 minutes at 80°C and then placed on the slides under a coverslip. Hybridization was performed in a sealed moist chamber at 37°C for 12 hours. The slides were then rapidly washed in 2XSSC with 50% formamide at 49°C and then 3 × 10 minutes in 2XSSC with 50% formamide at 39°C, 3 × 10 minutes with 2XSSC, and 2 × 30 minutes in 0.1% SSC at room temperature. After the post-hybridization washes, the slides were incubated at 37°C for 30 minutes with an anti-digoxigenin rhodamine-conjugate sheep antibody (5 μg/ml) (Boehringer, Mannheim). The signals were amplified by a rhodamin conjugated anti-sheep antibody from mice (10 μg/ml; Jackson Immunoresearch). The slides were counterstained with DAPI (4’, 6’- 266 diamidino-2- fenilindole) in 4XSSC and mounted in DABCO (1,4- diazabicyclo 2.2.2 octane) antifade. Hybridization signals and DAPI fluorescence were viewed using an Axioplan (Zeiss, Jena) microscope equipped with a cooled Charge-Coupled Device (CCD) camera (Digital Pixel Inc., Brighton). Digital images were pseudo-colored and merged, allowing for the simultaneous detection of hybridization signals and DAPI chromosome counterstaining. IPLab Spectrum version 3.1.1 software with the FISH-capture extension (Digital Scientific) was used to normalize and enhance the images.

### Availability of supporting data

Sequence data have been submitted to Genbank: accession numbers of the *Tubifex tubifex* and *Tubifex blanchardi* embryos, JQ247437-JQ247511. The Genbank accession numbers of the 16S rRNA and COI gene fragments of the 106 *Tubifex tubifex* and *Tubifex blanchardi*, 2 *Limnodrilus hoffmeisteri* and 2 *Potamotrix bavaricus* specimens are as follows: EU117465-EU117509, EU117546-EU117547, EU311221- EU311273, EU311275-EU311285 (16S); EU311286-EU311382, EU311384-EU311398 (COI).

## Competing interests

All contributing authors declare no conflict of interest.

## Authors’ contributions

RM, AC, ER and MF designed the experiments and analyzed and interpreted the data. RM, CF and ER performed the cytogenetic analyses. AC performed the phylogenetic analyses and RM and AC wrote the manuscript. All authors read an approved the final manuscript.

## Supplementary Material

Additional file 1: Figure S1 Neighbor-joining tree of the 16S rRNA gene fragment sequences of the *Tubifex *spp. used in this study and from [23], corresponding to the lineages with different cadmium resistances. Note that sequence D6-U9, officially belonging to lineage II (GenBank accession number AJ225905), groups with the *Tubifex* of lineage 2b and two other haplotypes belonging to lineage III*.* Bootstrap support values are indicated in front of the nodes. Ch, chromosome number; Cl, clade; Li, lineage. Click here for file
