# Supplementary figures and images for: Alike but different: the evolution of the Tubifex tubifex species complex (Annelida, Clitellata) through polyploidization
Source: BMC Evol Biol. 2014 Apr 2;14:73. doi: 10.1186/1471-2148-14-73 (PMC4021366; doi:10.1186/1471-2148-14-73)

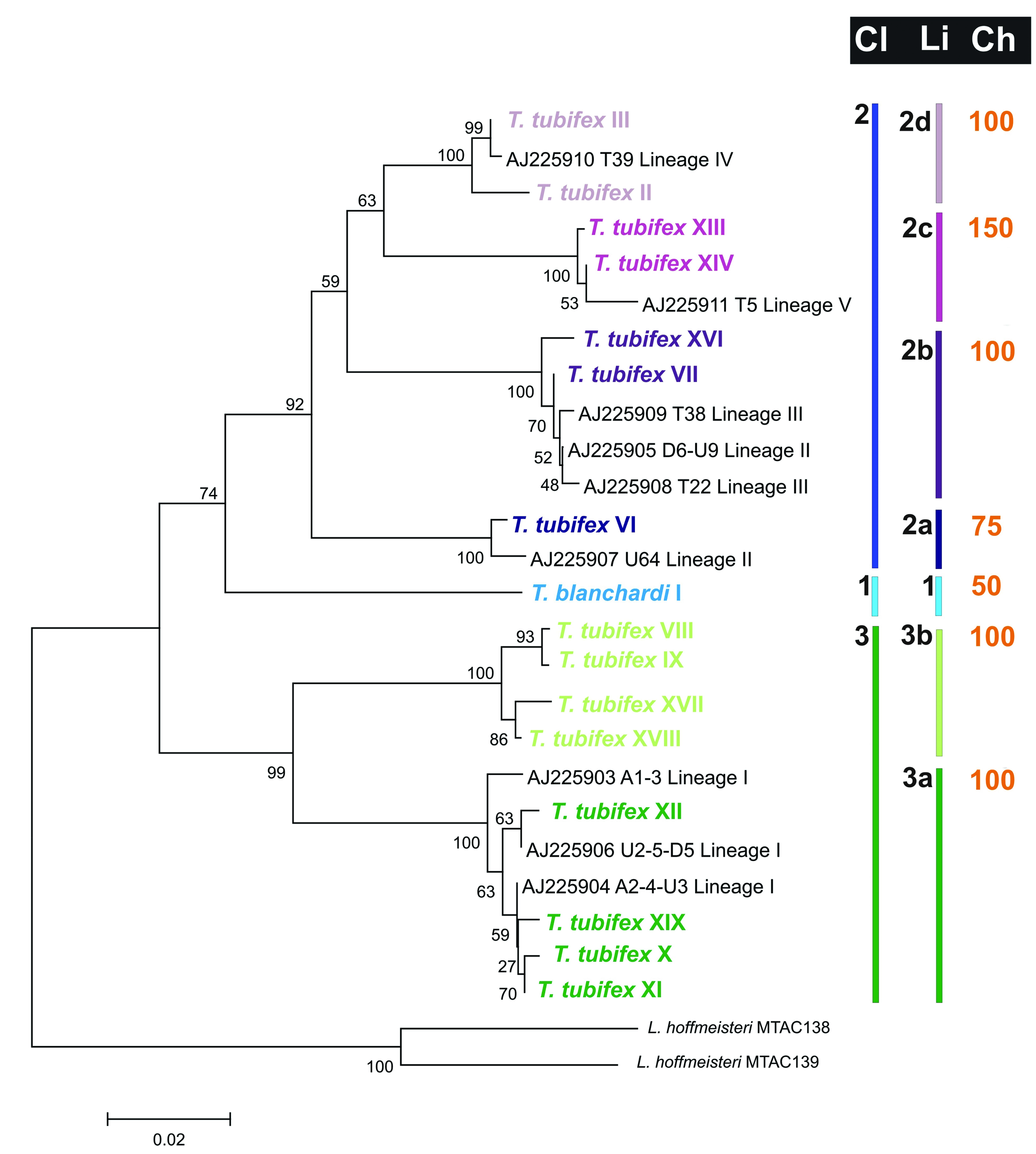

Supplement: Additional file 1: Figure S1 — Neighbor-joining tree of the 16S rRNA gene fragment sequences of the Tubifex spp. used in this study and from [23], corresponding to the lineages with different cadmium resistances. Note that sequence D6-U9, officially belonging to lineage II (GenBank accession number AJ225905), groups with the Tubifex of lineage 2b and two other haplotypes belonging to lineage III. Bootstrap support values are indicated in front of the nodes. Ch, chromosome number; Cl, clade; Li, lineage. [file 1471-2148-14-73-S1.tiff]
